# Case Report: Sebaceous lymphadenocarcinoma arising in parotid gland: eighth documented case with lymph node metastasis

**DOI:** 10.3389/fonc.2025.1639617

**Published:** 2025-12-01

**Authors:** Lu Ru, Fengxian An, Bo Zou

**Affiliations:** 1 Department of Oral and Maxillofacial Surgery, Liaocheng People’s Hospital, Medical School of Liaocheng University, Liaocheng, Shandong, China; 2 Department of Pathology, Liaocheng People’s Hospital, Medical School of Liaocheng University, Liaocheng, Shandong, China

**Keywords:** sebaceous lymphadenocarcinoma, parotid gland, sebaceous lymphadenoma, lymph node metastasis, case report

## Abstract

**Background:**

Sebaceous lymphadenocarcinoma (SLAC) represents an extremely rare parotid malignancy, hypothesized to originate through malignant transformation of sebaceous lymphadenoma. To date, fewer than ten histopathologically confirmed cases have been documented globally.

**Case summary:**

A 33-year-old female presented with a persistent, painless left parotid mass that persisted despite anti-infective therapy. The patient underwent a comprehensive diagnostic workup prior to surgical resection. Histopathological examination of the specimen revealed sebaceous lymphadenoma with distinct foci of malignant transformation to SLAC, confirming a localized malignant process. Based on a review of the literature, this case represents the eighth reported instance of SLAC and the third with pathologically confirmed lymph node metastasis.

**Conclusion:**

SLAC most commonly presents as a painless parotid mass. Surgical resection is the cornerstone of therapy, with individualized use of radiotherapy. While early outcomes may be favorable, long-term surveillance is essential. International collaboration is urgently needed to establish registries and define standardized management guidelines for this exceedingly rare malignancy.

## Introduction

Sebaceous lymphadenocarcinoma (SLAC) is an exceptionally rare, high-grade malignancy arising from ectopic sebocytes within lymphoid tissue (1). Characterized by dual sebaceous-lymphoid differentiation, this entity presents significant diagnostic and therapeutic challenges. Clinically, it typically manifests as a rapidly progressing mass, frequently accompanied by pain or discomfort (2). Involvement of the parotid gland is exceedingly rare, with only seven histopathologically confirmed cases documented in the literature to date. This scarcity of published evidence compounds the challenges in both diagnosis and clinical management. Herein, we report a case of parotid SLAC and analyze its clinicopathological characteristics, diagnostic modalities, and management.

## Case report

A 33-year-old female presented to our institution for evaluation of a persistent, slowly enlarging left subauricular mass with a one-month history. The lesion demonstrated progressive growth during this period. The patient had undergone a two-week course of empirical antibiotic therapy at an external facility, which failed to alleviate symptoms, and local tenderness persisted.

The patient denied any significant personal or family history of salivary gland tumors, cutaneous malignancies, or sebaceous lesions. No relevant occupational exposures or previous dermatological conditions suggesting a metastatic origin were identified.

Systemic evaluation revealed no evidence of an alternative primary malignancy, confirming the solitary parotid origin of the mass.

### Physical examination

Physical examination revealed a well-demarcated, firm mass approximately 4 cm in diameter located inferior to the left auricle. The lesion demonstrated circumscribed margins, restricted mobility, and slight tenderness on palpation. No clinical signs of facial nerve dysfunction were observed, and the parotid duct orifice showed no abnormal discharge.

### Imaging examinations

#### Color doppler ultrasound

**Figure 1:**
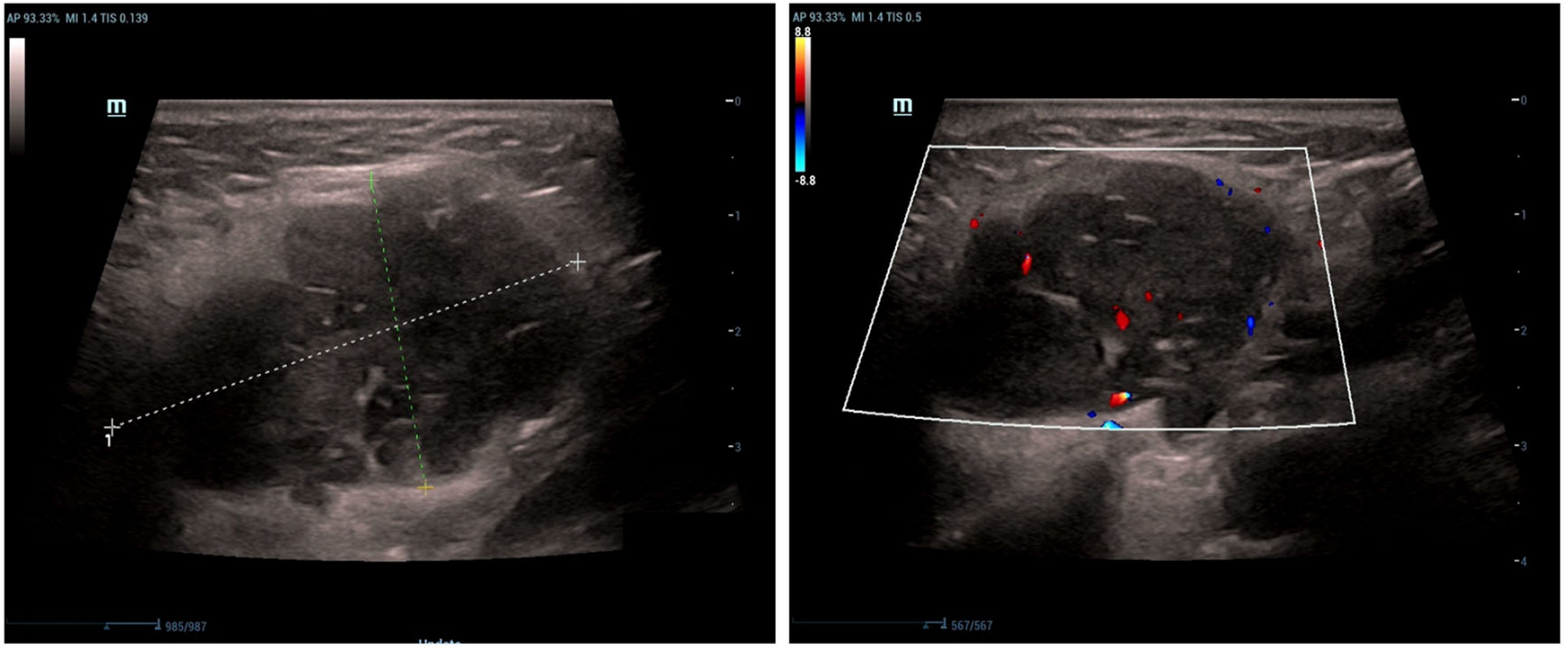
Longitudinal color Doppler ultrasound (12 MHz linear transducer) of the left parotid gland shows a hypoechoic lesion with irregular margins, heterogeneous echotexture, and internal vascularity.

The left parotid gland demonstrated a hypoechoic lesion measuring approximately 4.5 × 3.9 × 2.3 cm. This lesion exhibited circumscribed margins, an irregular contour, and heterogeneous internal echogenicity, with a distinct hyperechoic linear band visualized within it. Color Doppler Flow Imaging (CDFI) revealed strip-like vascularity within the lesion. Additionally, multiple lymph nodes were observed within and surrounding the left parotid parenchyma. The largest lymph node measured approximately 2.9 × 2.3 × 2.1 cm, with discernible borders but an indistinct corticomedullary differentiation. CDFI showed sparse intranodal vascularity (**Figure 1**).

#### Enhanced computed tomography

**Figure 2:**
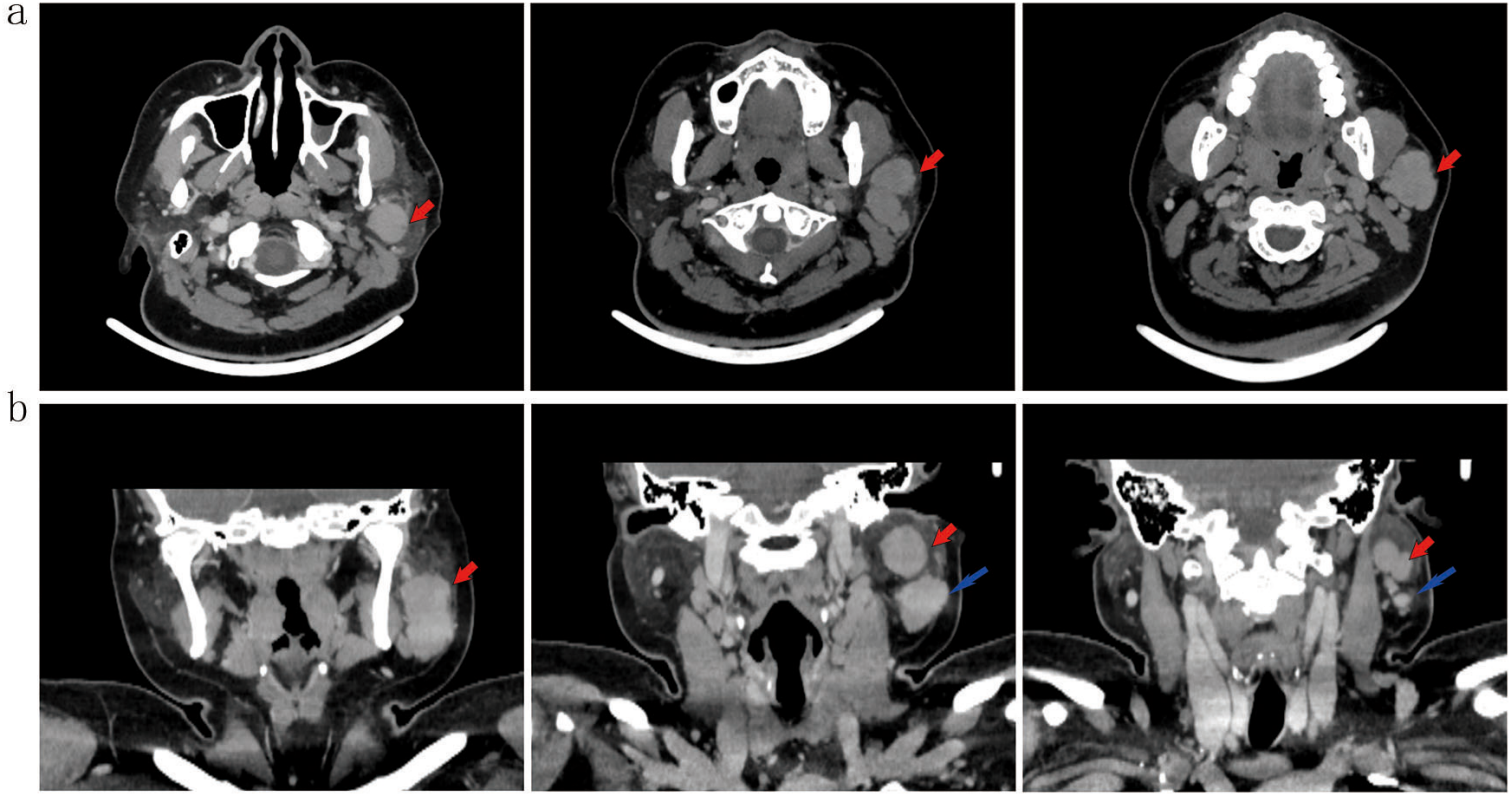
Axial **(a)** and coronal **(b)** contrast-enhanced CT(2.5 mm slice; IV iohexol 100 mL) shows a well-defined, lobulated soft-tissue lesion (red arrows; 3.6 × 3.0 × 4.1 cm) with moderate enhancement (~85 HU) in the left parotid gland. An adjacent lymph node is noted (blue arrow).

CT imaging identified multiple soft-tissue attenuation lesions within the left parotid gland. The dominant lesion measured approximately 3.6 × 3.0 × 4.1 cm. Following contrast administration, the lesion demonstrated moderate enhancement, with a post-contrast Hounsfield Unit (HU) value approximating 85 HU. The lesion margins were well-defined, exhibiting shallow lobulation (**Figure 2**).

### Preliminary diagnosis

Mass within the left parotid gland.

### Treatment

Under general anesthesia, a superficial parotidectomy was performed. The tumor was meticulously excised with a margin of surrounding parotid tissue while preserving the integrity of the facial nerve. An intraoperative frozen section consultation was conducted, which was assessed by an experienced pathologist. The frozen sections were consistent with a low-grade malignant neoplasm. Based on this critical intraoperative finding, we proceeded with a selective neck dissection (Levels I-III) to address potential regional metastasis. The postoperative course was uneventful, with no significant complications such as facial nerve weakness or Frey’s syndrome observed.

### Histopathological assessment

Two tumor nodules, measuring 5.8 × 3.8 × 3.0 cm and 4.0 × 2.4 × 2.4 cm, were identified within a prominent lymphatic stroma. The neoplastic cells were arranged in nests and sheets and demonstrated mild to moderate nuclear atypia, conspicuous nucleoli, discernible mitotic activity, and infiltrative growth. No definitive vascular invasion or perineural infiltration was identified. All surgical margins were free of tumor.

### Immunohistochemistry

**Figure 3:**
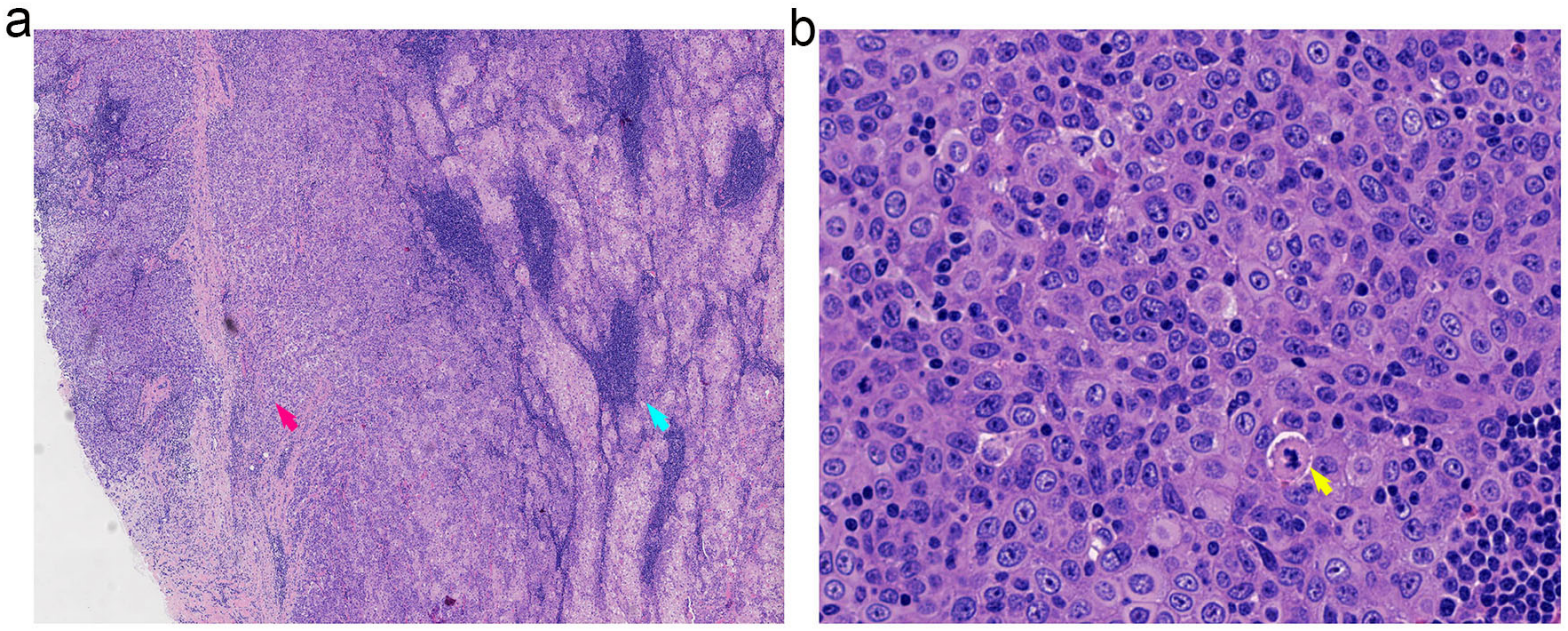
Histopathological features of SLAC demonstrating the transition from benign to malignant components. **(a)** Low-power view (20x magnification) illustrating the distinct transition zone between benign sebaceous structures (right, blue arrow) and infiltrating malignant carcinoma (left, red arrow). **(b)** High-power view (40x magnification) of the malignant component revealing cytological atypia with notable nuclear pleomorphism and atypical mitotic figures.

Tumor cells were diffusely positive for P40, focally positive for P16, and showed patchy (5%) expression of p53. The Ki-67 proliferation index was 20%. There was strong positivity for pan-cytokeratin (AE1/AE3). Staining for PRAME, S-100, EMA, and androgen receptor (AR) was partially positive. CD5 and BCL-2 highlighted background lymphocytes. The tumor was negative for CD117, CK7, NUT (clone C52B1), and SOX10. EBER *in situ* hybridization was negative (**Figure 3**, **Supplementary Table 1**).

### Final diagnosis

The findings supported a final diagnosis of sebaceous lymphadenocarcinoma (SLAC).

### Follow-up

The patient remained free of local recurrence and distant metastasis throughout the 12-month surveillance period.

## Discussion

SLAC represents an exceptionally rare malignant neoplasm arising primarily in the head and neck region, with the parotid gland being the predominant site of occurrence (1). Its precise incidence remains undefined, as fewer than ten histologically confirmed cases have been documented worldwide. A comprehensive review of the seven previously reported cases reveals a male predilection (male-to-female ratio: 5:2) and predominantly elderly presentation, with six patients aged >50 years at diagnosis and only one aged <50 years (1–7). Notably, the current case—occurring in a 33-year-old female—establishes the youngest recorded age of onset for parotid SLAC to date. The documented disease duration across cases ranges from 1 month to 20 years. Clinically, SLAC typically presents as a progressively enlarging, asymptomatic mass; however, three cases demonstrated accelerated growth within a short timeframe. One exceptional case initially manifested with cheek skin erythema. Surgical resection constituted the primary therapeutic approach in all patients, with five receiving adjuvant radiotherapy. During follow-up (duration range: 12–240 months), no tumor recurrence or disease-specific mortality was observed. Two elderly patients died of unrelated causes (cardiovascular disease and respiratory failure, respectively; **Table 1**).

**Table 1:** Previously reported cases of sebaceous adenocarcinoma.

Ref.	Age	Sex	History	Clinical manifestations	Histology	Treatment	Follow up
Linhartová A, et al. (7)	68	Female	2.5 years	Painless mass, Gradually increasing	Sebaceous carcinoma arising from lymphadenom	Excision and postoperative radiotherap	NER at 6 yr
Gnepp, DR, et al. (6)	70	Male	20 years	Painless mass	Poorly differentiated carcinoma arising from lymphadenoma	Excision and superficial parotidectomy	Died of cardiovascular disease at 18 mo
Gnepp DR. et al (5)	70	Male	1 mo	Painless mass	Salivary duct carcinoma with focal epithelial– myoepithelial carcinoma arising from lymphadenoma	Excision and superficial parotidectomy	NER at 14 mo
Croitoru1 et al. (2)	55	Male	3 years	Painless mass with recent rapid enlargement	Sebaceous carcinoma arising from lymphadenoma	Excision and postoperative radiotherapy	NER at 4 mo
Ahn, SH (4)	36	Male	10 years	Painless mass with recent rapid enlargement	Sebaceous carcinoma arising from lymphadenoma	Excision and postoperative radiotherapy	NER at 2 year
Claudius, K (3)	87	Male	2–6 mo	Painless mass,erythema of the cheek skin	Sebaceous carcinoma arising from lymphadenoma	Excision and postoperative radiotherapy	Died of respiratory failure at 8 mo
Hao, FY (1)	82	Female	4 mo	Painless mass with recent rapid enlargement	Sebaceous carcinoma arising from lymphadenoma	Excision and postoperative radiotherapy	NER at 10 mo

NER, No evidence of recurrence.

According to the 2022 WHO 5th Edition Classification of Salivary Gland Tumors, SLAC is categorized as a malignant epithelial tumor within the specific subgroup of sebaceous gland tumors (8). This subgroup encompasses sebaceous adenoma, lymphadenoma, carcinoma, lymphadenocarcinoma, and other neoplasms exhibiting sebaceous differentiation. Current evidence indicates that SLAC typically arises from the malignant transformation of benign precursors, particularly sebaceous lymphadenoma (SLA). Notably, all seven previously reported SLAC cases arose from pre-existing SLA, yet they exhibited considerable heterogeneity in their malignant components. Specifically, five cases were classified as sebaceous carcinoma, one as a poorly differentiated carcinoma, and one as a salivary duct carcinoma with focal epithelial-myoepithelial carcinoma features. Similarly, our case presented as a histologically confirmed sebaceous carcinoma arising within an SLA.

SLA, a rare benign tumor of the major salivary glands, was first characterized by McGravan et al. in 1960 (9). An earlier analogous lesion in the parotid gland reported by Rawson et al. (24) is now classified as non-sebaceous lymphadenoma (10). These entities are pathologically distinguished by the presence or absence of sebaceous differentiation within the lymphoid stroma (11). As a benign neoplasm, SLA is typically cured by complete surgical excision and exhibits minimal recurrence risk. It predominates in patients >50 years without significant gender predilection. When arising in salivary glands, SLA most commonly occupies the superficial parotid lobe. Rare occurrences have been documented in the maxilla (12), minor salivary glands of the lower lip (13), and occasionally synchronously with Warthin tumor (adenolymphoma) (14). Radiologically, SLA appears as heterogeneous solid nodules on CT/MRI, often demonstrating multifocal cystic components and characteristic fatty infiltration that may yield a T1-hyperintense signal (15).

Imaging is pivotal in the initial assessment of parotid masses. In our case and as reported in the literature (15), ultrasound of sebaceous lymphadenoma and its malignant counterpart typically reveals a circumscribed, hypoechoic lesion with heterogeneous echotexture. Cystic spaces and hyperechoic linear bands may reflect sebaceous elements and fibrous septa, respectively. On contrast-enhanced CT, such lesions generally present as well-defined, lobulated masses with moderate enhancement. Areas of low attenuation can indicate cystic change or fatty infiltration—a hallmark of sebaceous differentiation. Although suggestive, these features are not pathognomonic for malignancy; however, the coexistence of enlarged lymph nodes with effaced corticomedullary architecture, as seen here, should raise strong suspicion for malignant transformation and nodal metastasis.

The precise molecular drivers underlying the malignant transformation of SLA into SLAC remain elusive (16). Histologically, this transformation is characterized by invasive growth of malignant cell nests within a benign background—comprised of characteristic vacuolated sebocytes, lymphocytes, and basaloid cells (17). Although limited evidence suggests a potential role of Epstein-Barr virus (EBV) in this process, with EBER positivity reported in previously documented cases (18), the current case demonstrated unequivocal EBER negativity. Furthermore, our immunohistochemical results provide additional evidence supporting pathogenic heterogeneity. The focal AR expression (present in 10% of tumor cells) suggests the presence of a salivary duct carcinoma-like subpopulation arising during malignant progression, although this does not represent the dominant phenotype. The complete absence of PRAME staining argues against a highly aggressive tumor biology, which is generally correlated with strong, diffuse PRAME expression. Likewise, the focal and non-diffuse p16 staining pattern (30% positivity) is inconsistent with HPV-related carcinogenesis and instead implies alternative mechanisms of cell cycle dysregulation, such as alterations in the RB pathway. Collectively, these observations enhance our understanding of SLAC pathogenesis and further indicate heterogeneity in its underlying molecular mechanisms.

The histopathological complexity and dual differentiation of SLAC create significant diagnostic challenges, necessitating its distinction from several more common parotid neoplasms. Warthin’s tumor shares a lymphoid stroma but is distinguished by its benign oncocytic papillary epithelium, lacking sebaceous differentiation and malignancy (19).

Pleomorphic adenoma lacks the dense lymphoid background and sebaceous cells, featuring instead a characteristic chondromyxoid stroma (20). Mucoepidermoid carcinoma (MEC) contains mucinous and epidermoid cells; even rare sebaceous variants lack the organoid lymphoid stroma of SLAC (21). Metastatic cutaneous sebaceous carcinoma is ruled out primarily by the absence of a skin primary and the presence of a benign SLA component, which is absent in pure metastasis (22). Lymphoepithelial carcinoma (LEC) is EBV-driven (EBER+), poorly differentiated, and lacks sebaceous differentiation (18). Salivary duct carcinoma (SDC) exhibits diffuse strong AR and HER2 positivity, comedo-necrosis, and lacks a lymphoid background (23). The immunoprofile of our case (p40+, AR focal+, EBER-, p16 non-diffuse+) is pivotal, helping exclude LEC (EBER+), HPV-related carcinomas (p16+), and classic SDC (AR diffuse+).

The diagnostic utility of fine-needle aspiration cytology (FNAC) in SLAC is markedly limited. Aspirates are often dominated by a prominent benign lymphoid population and mature sebaceous cells, frequently leading to an erroneous interpretation of a benign or inflammatory process—such as chronic sialadenitis, reactive lymphadenopathy, or SLA. The sparse representation of malignant epithelial cells in the sample is readily overlooked, particularly in the absence of high clinical suspicion. Thus, although FNAC can confirm a mass lesion, it is generally unreliable for a definitive preoperative diagnosis of SLAC. Consequently, complete surgical excision followed by comprehensive histopathological assessment and immunohistochemical profiling remains essential for accurate diagnosis.

The mainstay of treatment for SLAC is surgical resection, with adjuvant radiotherapy considered based on pathological risk factors such as nodal involvement or close margins. This combined approach has been associated with favorable oncological outcomes in previously reported cases (1). In the present case, the patient underwent complete surgical resection. Although postoperative radiotherapy was recommended due to the presence of cervical lymph node metastasis, the patient declined this adjuvant treatment due to quality-of-life concerns. This decision was made through a shared decision-making process that balanced the uncertain survival benefit of radiotherapy against its well-established long-term toxicities—a particularly relevant consideration for this young patient. The care plan respected the patient’s prioritization of fertility preservation and functional outcomes, and an intensified surveillance protocol was implemented to mitigate potential risks. This approach highlights that management of ultra-rare malignancies often requires individualized strategies that extend beyond standardized algorithms.

Given the absence of established guidelines, our surveillance strategy was designed based on a risk-adapted framework (1): high-frequency MRI (quarterly for the first 2 years) to detect local recurrence during the period of greatest risk (2); subsequent transition to biannual low-dose CT combined with ultrasound for intermediate-term monitoring (years 3-5) to balance detection sensitivity and long-term radiation safety; and (3) lifelong annual ultrasound screening to account for the unpredictable biology of SLAC. The patient remains under this active surveillance protocol, and no evidence of recurrence has been observed during the 12-month follow-up period. However, long-term monitoring remains imperative. The extreme rarity of SLAC continues to pose significant challenges for conducting meaningful clinical studies; therefore, overcoming these challenges will require unprecedented international collaboration.

## Conclusion

SLAC typically presents as a painless parotid mass. Complete surgical resection is the primary treatment, with neck dissection and adjuvant radiotherapy guided by individual risk assessment. Despite often favorable early outcomes, long-term vigilance is essential due to its rarity and unpredictable behavior. Future efforts should focus on establishing an international, multi-institutional registry to aggregate cases and facilitate collaborative research. Larger case series and molecular studies are imperative to better elucidate the tumor’s biology, refine prognostic predictors, and ultimately establish evidence-based treatment guidelines.

## Data Availability

The raw data supporting the conclusions of this article will be made available by the authors, without undue reservation.
