# Supplementary material for: Case Report: Sebaceous lymphadenocarcinoma arising in parotid gland: eighth documented case with lymph node metastasis
Source: Front Oncol. 2025 Dec 1;15:1639617. doi: 10.3389/fonc.2025.1639617 (PMC12702721; doi:10.3389/fonc.2025.1639617)
Supplement: Supplementary file 1 [file Table1.docx]

**S****upplementary Table 1. Detailed Immunohistochemistry Results**

| **Antiboy** | **Staining Pattern** | **Intensity** | **Positivity (%)** |
| --- | --- | --- | --- |
| **P40** | Nuclear | +++ | 90% |
| **P16** | Nuclear/Cytoplasmic | ++ | 30% |
| **P53** | Nuclear | + | 5% |
| **Ki-67** | Nuclear | +++ | 20% |
| **CK(AE1/AE3)** | Cytoplasmic | +++ | 100% |
| **AR(Androgen Receptor)** | Nuclear | ++ | 10% |
| **PRAME** | Nuclear/Cytoplasmic | - | 0 |
| **S-100** | Nuclear/Cytoplasmic | +++ | 10% |
| **EMA** | Membranous/Cytoplasmic | +++ | 90% |
| **CD5** | Membranous | +++ | T-lymphocytes |
| **BCL-2** | Cytoplasmic | +++ | T-lymphocytes |
| **CD117** | - | 0 | 0% |
| **CK7** | - | 0 | 0% |
| **SOX10** | - | 0 | 0% |
| **NUT (C52B1)** | - | 0 | 0% |
| **EBER (ISH)** | - | 0 | 0% |

Staining Pattern (e.g., Nuclear, Cytoplasmic, Membranous); Staining Intensity (scored as 0: Negative; 1+: Weak; 2+: Moderate; 3+: Strong); Percentage Positivity (%);
